# Influenza Epidemiology and Vaccine Effectiveness among Patients with Influenza-Like Illness, Viral Watch Sentinel Sites, South Africa, 2005–2009

**DOI:** 10.1371/journal.pone.0094681

**Published:** 2014-04-15

**Authors:** Genevie M. Ntshoe, Johanna M. McAnerney, Stefano Tempia, Lucille Blumberg, Jocelyn Moyes, Amelia Buys, Dhamari Naidoo, Marietjie Venter, Terry Besselaar, Barry D. Schoub, Bernice N. Harris, Cheryl Cohen

**Affiliations:** 1 Division of Public Health Surveillance and Response, National Institute for Communicable Diseases of the National Health Laboratory Service, Johannesburg, South Africa; 2 Centre for Respiratory Diseases and Meningitis, National Institute for Communicable Diseases of the National Health Laboratory Service, Johannesburg, South Africa; 3 Influenza Division, United States Centers for Disease Control and Prevention, National Institute for Communicable Diseases of the National Health Laboratory Service, Johannesburg, South Africa; 4 World Health Organisation, Geneva, Switzerland; 5 Centre for Vaccines and Immunology, National Institute for Communicable Diseases of the National Health Laboratory Service, Johannesburg, South Africa; 6 School of Health Systems and Public Health, University of Pretoria, Pretoria, South Africa; University of Hong Kong, Hong Kong

## Abstract

**Background:**

There is limited data on the epidemiology of influenza and few published estimates of influenza vaccine effectiveness (VE) from Africa. In April 2009, a new influenza virus strain infecting humans was identified and rapidly spread globally. We compared the characteristics of patients ill with influenza A(H1N1)pdm09 virus to those ill with seasonal influenza and estimated influenza vaccine effectiveness during five influenza seasons (2005–2009) in South Africa.

**Methods:**

Epidemiological data and throat and/or nasal swabs were collected from patients with influenza-like illness (ILI) at sentinel sites. Samples were tested for seasonal influenza viruses using culture, haemagglutination inhibition tests and/or polymerase chain reaction (PCR) and for influenza A(H1N1)pdm09 by real-time PCR. For the vaccine effectiveness (VE) analysis we considered patients testing positive for influenza A and/or B as cases and those testing negative for influenza as controls. Age-adjusted VE was calculated as 1-odds ratio for influenza in vaccinated and non-vaccinated individuals.

**Results:**

From 2005 through 2009 we identified 3,717 influenza case-patients. The median age was significantly lower among patients infected with influenza A(H1N1)pdm09 virus than those with seasonal influenza, 17 and 27 years respectively (p<0.001). The vaccine coverage during the influenza season ranged from 3.4% in 2009 to 5.1% in 2006 and was higher in the ≥50 years (range 6.9% in 2008 to 13.2% in 2006) than in the <50 years age group (range 2.2% in 2007 to 3.7% in 2006). The age-adjusted VE estimates for seasonal influenza were 48.6% (4.9%, 73.2%); −14.2% (−9.7%, 34.8%); 12.0% (−70.4%, 55.4%); 67.4% (12.4%, 90.3%) and 29.6% (−21.5%, 60.1%) from 2005 to 2009 respectively. For the A(H1N1)pdm09 season, the efficacy of seasonal vaccine was −6.4% (−93.5%, 43.3%).

**Conclusion:**

Influenza vaccine demonstrated a significant protective effect in two of the five years evaluated. Low vaccine coverage may have reduced power to estimate vaccine effectiveness.

## Introduction

Influenza is an acute viral infection characterized by rapid spread, regular winter epidemics in temperate countries and year-round circulation in the tropical regions [1]–[2]. It is highly infectious and associated with significant morbidity and mortality in high-risk individuals worldwide [3]. In South Africa, influenza and pneumonia were the second leading cause of death during the years 2005 to 2009 [4]–[8].

The global influenza surveillance network of the World Health Organization (WHO) serves as a mechanism to monitor the influenza types and subtypes circulating globally as well as an alert mechanism for the emergence of novel influenza viruses with potential to cause pandemics [9]. In April 2009, a new influenza virus strain infecting humans was detected in the United States and Mexico and by 15 July had spread to more than 100 countries including South Africa [10]–[11].

Vaccination is the primary public health measure for preventing influenza infection [3]. However, circulating influenza viruses constantly change requiring an annual update of influenza vaccines to match the current circulating strains [12]. Twice yearly, the WHO recommends the content of the influenza vaccine for the forthcoming influenza season [9]. These recommendations are based on data submitted by its global influenza surveillance network [9]. In South Africa, influenza vaccination is provided at no charge at public health facilities for people who are at risk of severe disease (persons aged >65 years, those with underlying conditions, pregnant women, residents of rehabilitation institutions, children on long-term aspirin therapy, healthcare workers responsible for the care of high risk cases, family contacts of high-risk cases) and is available at a fee in the private sector [13]. During 2011–2013 influenza seasons, vaccine coverage among people aged >65 years and pregnant women were reported to be 2% and 14% respectively (Ramkrishna W *et al* – Options for the Control of Influenza VIII).

Prior to 2006, there was limited surveillance for influenza and little was known about the epidemiology of influenza on the continent. However progress has been made in recent years [2], [14]–[15]. Nevertheless, there are still limited data on the uptake of influenza vaccines and their effectiveness on the African continent. Several studies in other parts of the world have shown the feasibility of estimating vaccine effectiveness (VE) from surveillance data [16]–[17]. We analysed influenza-like illness (ILI) surveillance data to compare the epidemiological characteristics of patients infected with influenza A(H1N1)pdm09 virus to those infected with seasonal influenza. In addition we estimated influenza vaccine effectiveness (VE) from the national ILI surveillance network during five influenza seasons (2005–2009) and assessed whether the 2009 seasonal influenza vaccine had a protective effect against the A(H1N1)pdm09 influenza virus in the same year. During the period of our study only seasonal influenza vaccine was available and there was no pandemic vaccine available.

## Methods

### Ethics Statement

The NICD has ethics clearance for essential communicable disease surveillance activities of public health importance in South Africa granted by the Human Medical Research Ethics Committee of the University of the Witwatersrand, Johannesburg. Our study was conducted using surveillance data that fall into the specification mentioned above. None of the authors participated in sample collection. Samples were given a unique identifier before analysis. If requested our data will be made available upon publication.

### Study Design and Setting

The Viral Watch in South Africa is a prospective influenza surveillance programme based on a network of sentinel general practitioners who report on ILI cases seen in their practices [18]. It is coordinated by the National Institute for Communicable Diseases (NICD) of the National Health Laboratory Service (NHLS). The programme encompasses mainly (approximately 90%) private primary health care centers and some public facilities situated in all nine provinces of South Africa and is conducted throughout the year [18]. It is estimated that 16% of individuals in South Africa seek care in the private sector [19]. In 2005 the sentinel sites were situated only in Gauteng Province. From 2006 the surveillance programme progressively expanded to reach coverage in all nine provinces in 2008 [18]. During the study period (2005–2009), demographic characteristics, date of illness onset and sample collection, signs and symptoms (data on underlying condition were not available), and influenza vaccination history were collected using standard data collection forms from patients who presented with ILI defined as sudden onset of fever (temperature of ≥38°C) with at least two of the following symptoms: cough, headache, myalgia or sore throat. Sample collection was recommended to be within three days of onset of symptoms; however a small proportion (10%) of specimens collected >3 days after onset were also received [18]. An ILI case was defined as influenza positive when laboratory results positive for influenza A and/or B viruses were obtained. In 2009, because of the increased demand for laboratory testing with the advent of influenza A(H1N1)pdm09 in South Africa, sentinel sites were requested to limit the number of enrolled cases to a maximum of five per week. In the previous years there was no limitation on enrollment of cases.

### Sample Collection and Laboratory Testing

Throat and/or nasal swabs were collected from all enrolled patients and transported (on ice) to the laboratory in viral transport medium (VTM) for influenza virus detection. Specimens from seven of the nine provinces were tested at the National Influenza Centre (NIC) situated at the NICD-NHLS, while specimens from two other provinces (KwaZulu-Natal and Western Cape) were tested at their respective laboratories and positive samples sent to the NICD-NHLS for subtyping and sequencing. From 2005–2007, typing was performed mainly by haemagglutination inhibition (HAI) test while in 2008 about 50% could not be typed by HAI hence PCR was used. In 2009, due to the emerging pandemic strain, the more sensitive United States Centers for Disease Control and Prevention polymerase chain reaction (PCR) was used for the detection and characterization of the influenza A(H1N1)pdm09 virus. Few HAI tests were performed in 2009.

### Data Management and Analysis

The detection rate (number of influenza positive specimens/number of specimens submitted) was calculated only for those specimens tested at the NICD during the influenza season as we received very low numbers during the influenza off-season (although clinicians are requested to submit specimens throughout the year) which can lead to falsely high detection rates.

For the VE analysis, patients were considered vaccinated if they received influenza vaccine ≥2 weeks before onset of symptoms and unvaccinated if they were not vaccinated for that season or received influenza vaccine <2 weeks before symptoms onset. Patients without reported vaccination status or vaccination date were classified as unknown and were excluded from the VE analysis. We restricted the VE analysis to patients who presented at Viral Watch sentinel sites from seven of the nine provinces within the influenza season. We did not have denominators of specimens tested in the other two provinces as a result they were excluded from the VE analysis.

The start and the end of the influenza season were defined as a weekly detection rate of ≥10% and <10% for two consecutive weeks respectively. In 2009 two distinct waves of influenza circulation were observed, the first was dominated by influenza A(H3N2) and the second by influenza A(H1N1)pdm09. There was a two week overlap between the two waves, as a result specimens collected during this period were classified in both seasons. However, patients who tested positive for A(H1N1)pdm09 virus (n = 161) during the 2009 seasonal influenza were considered as negative for seasonal influenza while those who tested positive for seasonal influenza viruses (n = 129) during the A(H1N1)pdm09 virus circulation were considered as negative for A(H1N1)pdm09 virus in the VE analysis.

We considered patients with ILI and positive for influenza A and/or B viruses as cases and patients with ILI, but laboratory-negative for influenza as controls [20]–[21]. Age-adjusted (<50 and ≥50 years) VE was calculated as 1-odds ratio (OR) for influenza in vaccinated and non-vaccinated individuals. Significance was assessed at p<0.05 for all analysis. Data were analyzed using OpenEpi version 2.3 (US Centers for Disease Control and Prevention, Atlanta, Georgia, United States).

## Results

### Influenza Virus Detection and Seasonality

**Figure 1:**
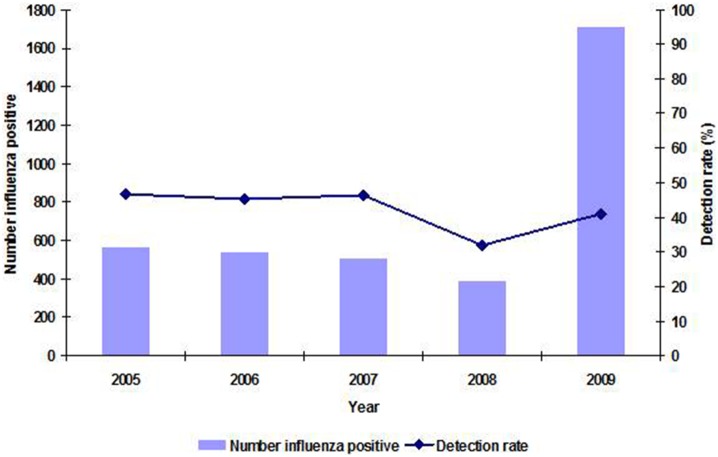
Number of specimens testing positive for influenza and detection rates by year, Viral Watch, South Africa, 2005–2009.

From 2005 through 2009, a total of 8,559 specimens were received at the NICD for the detection of respiratory viruses from Viral Watch sentinel sites. Of these, 3,205 (37%) tested positive for influenza A and/or B viruses. In addition another 512 positive specimens were received from KwaZulu-Natal and Western Cape provinces, bringing the total influenza positives to 3,717 (ranging from 388 influenza positives in 2008 to 1,714 in 2009– Figure 1). Of those, 3,248 (87%) were influenza A, 458 (12%) influenza B and 11 (0.3%) tested positive for both A and B influenza viruses. Of the 11 patients with both A and B influenza viruses identified, three were excluded from the analysis as both pandemic and seasonal influenza viruses were detected. The other eight mixed infections were classified as seasonal as the A influenza virus identified was the seasonal one. Among the influenza A viruses, 3,075 (95%) were further subtyped; 1,597 (52%) were A(H3N2), 776 (25%) were A(H1N1) and 702 (23%) were A(H1N1)pdm09 virus. The annual detection rate amongst specimens tested at NICD during the influenza seasons ranged from 32% in 2008 to 47% in 2005 and 2007 (Figure 1).

**Figure 2:**
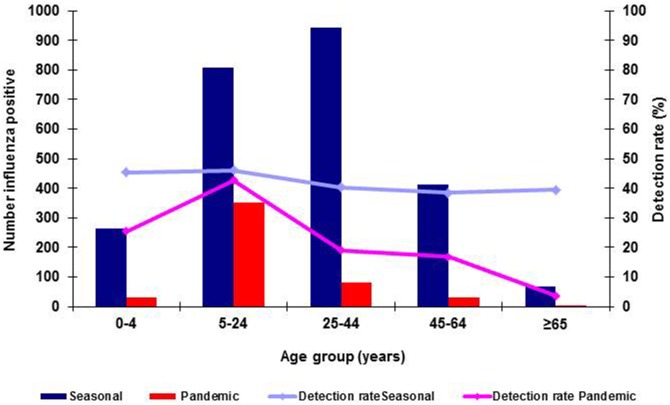
Influenza detections and detection rates for seasonal influenza (N = 2490) and influenza A(H1N1)pdm09 (N = 496) by age group, Viral Watch, South Africa: 2005–2009.

**Figure 3:**
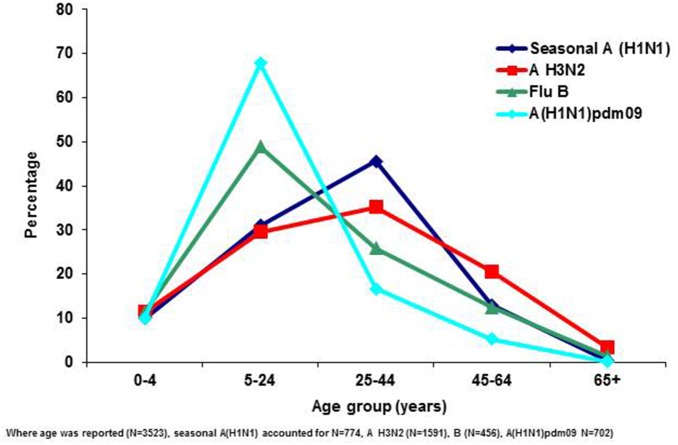
Percentage of influenza positives by age group and virus type, Viral Watch, South Africa; 2005–2009.

The median age was significantly lower among patients infected with influenza A(H1N1)pdm09 virus (17, range: 0–97 years) than those infected with seasonal influenza viruses (A(H1N1), A(H3N2) and B), (27, 0–84years) (p<0.001). However, the highest detection rate was among the 5–24 years age group for both pandemic and seasonal influenza cases (Figure 2). The age distribution of influenza A(H1N1)pdm09 case-patients was similar to those infected with influenza B but differed from that of patients with seasonal influenza A subtypes A(H1N1) and A(H3N2) (Figure 3). The median age for influenza A(H1N1)pdm09 and seasonal influenza B patients was 17 (range 0–97) and 18 (range 0–78) years respectively while those for seasonal A(H1N1) and A(H3N2) was 28 (range 0–73) and 29 (range 0–84) years respectively.

**Figure 4:**
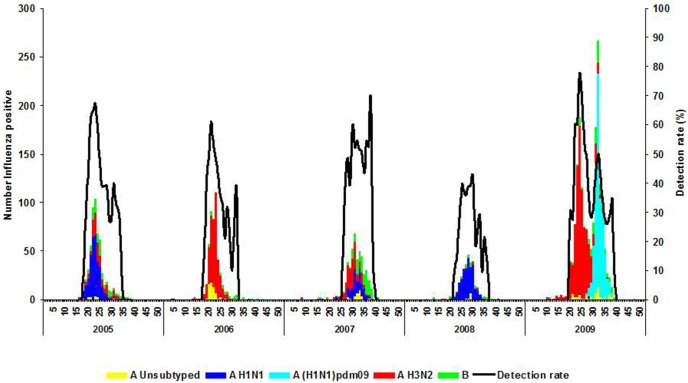
Number of influenza positives by virus type, subtype and detection rate by week and year, Viral Watch, South Africa, 2005–2009.

Predominant A subtypes differed by year. Influenza A(H1N1) was predominant during the years 2005 (317/564, 56%) and 2008 (308/388, 79%) while A(H3N2) was predominant during 2006 (417/541, 77%) and 2007 (208/510, 41%). From 2005 through 2008, influenza epidemics were unimodal and occurred predominantly from June to August. The duration of the seasonal influenza period ranged from 11 weeks in 2009 to 19 weeks in 2005. In 2009 two distinct waves of influenza circulation were observed: the first occurred in May to July and was dominated by influenza A(H3N2) virus and lasted for 11 weeks, while the second occurred in July to September and was dominated by influenza A(H1N1)pdm09 virus and lasted for a period of eight weeks (Figure 4).

### Vaccine Effectiveness

From 2005 through 2009 during the influenza season, a total of 7,535 ILI patients from seven of the nine provinces were enrolled as follows: 5,946 (79%) during five seasonal influenza seasons and 1,589 (21%) during the A(H1N1)pdm09 circulation period in 2009. Of the patients enrolled during the seasonal influenza season and the A(H1N1)pdm09 circulation period 95% (5,649/5,946) and 97% (1,541/1,589) met the ILI WHO case definition of fever ≥38°C and cough or sore throat, respectively. Two percent (140/5,946) and 6% (93/1,589) of cases during the seasonal and pandemic influenza period respectively were excluded from the VE analysis because of their unknown vaccination status. In 2009 influenza A(H3N2) (n = 129) and A(H1N1)pdm09 (n = 161) co-circulated for a period of two weeks.

#### Seasonal influenza

**Figure 5:**
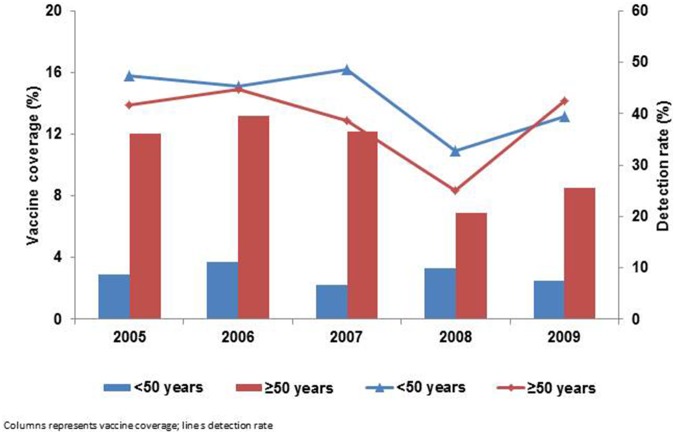
The vaccine coverage and detection rate during influenza by year and age group, Viral Watch, South Africa, 2005–2009.

Of the 5,806 patients enrolled during the pre-2009 pandemic influenza seasons and with known vaccination status, 2,502 (43%) tested positive for influenza A and/or B viruses and 234 (4%) received influenza vaccine. The overall vaccine coverage during the pre-2009 pandemic influenza seasons was 4.0% (range 3.4% in 2009 to 5.1% in 2006 (Table 1) and was higher in the ≥50 years (range 6.9% in 2008 to 13.2% in 2006) than in the <50 years (range 2.2% in 2007 to 3.7% in 2006) age groups in all five influenza seasons. The influenza detection rate ranged from 31.9% in 2008 to 46.7% in 2005 and was higher in the <50 years (range 32.8% in 2008 to 47.4% in 2005) than in the ≥50 years (range 25.0% in 2008 to 44.6% in 2006) in four (2005–2008) of the five influenza seasons (Figure 5).

**Table 1:** Comparison of vaccine composition to circulating viruses, South Africa, 2005–2009.

Year	Vaccine composition	Circulating viruses
2005	A/New Caledonia/20/99(H1N1)-like virus	**A/New Caledonia/20/99(H1N1)-like virus**
	A/Wellington/1/2004(H3N2)-like virus	A/California/7/2004(H3N2)-like virus
	B/Shanghai/361/2002-like virus	B/Hong Kong/333/01-like virus
2006	A/New Caledonia/20/99(H1N1)-like virus	A/New Caledonia/20/99(H1N1)-like virus
	A/California/7/2004(H3N2)-like virus	**A/Wisconsin/67/2005(H3N2)-like virus**
	B/Malaysia/2506/2004-like virus	B/Malaysia/2506/2004-like virus
2007	A/New Caledonia/20/99(H1N1)-like virus	A/Solomon Islands/3/2006 (H1N1)-like virus
	A/Wisconsin/67/2005(H3N2)-like virus	**A/Brisbane/10/2007 (H3N2)-like virus**
	B/Malaysia/2506/2004-like virus	B/Malaysia/2506/2004-like virus
2008	A/Solomon Islands/3/2006 (H1N1)-like virus	**A/Brisbane/59/2007 (H1N1)-like virus**
	A/Brisbane/10/2007 (H3N2)-like virus	A/Brisbane/10/2007 (H3N2)-like virus
	B/Florida/4/2006-like virus	B/Florida/4/2006-like virus
2009	A/Brisbane/59/2007 (H1N1)-like virus	**A/California/7/2009 (H1N1)-like virus**
	A/Brisbane/10/2007 (H3N2)-like virus	**A/Perth/16/2009 (H3N2)-like virus**
	B/Florida/4/2006-like virus	B/Brisbane like-virus

**Predominating circulating strains in bold.**

**Predominating B strains indicated.**

The age-adjusted VE estimates ranged from −14.2% (95% CI: −99.7% to 34.8%) in 2006 to 67.4% in 2008 (95% CI: 12.4% to 90.3%) (Table 1). Influenza vaccination demonstrated a significant protective effect during the 2005 (VE: 48.6%, 95%CI: 4.9 to 73.2) and 2008 (VE: 67.4%, 95%CI: 12.4 to 90.3) seasonal influenza seasons (Table 1). Stratifying by age, there was no significant difference between the two age groups except in 2005 where a higher VE of 73% (95% CI: 36.2 to 90.1) was noted in those aged <50 than in the ≥50 age group (VE: −29.3%, 95% CI: −245.3 to 52.1).

The vaccine composition and the influenza types circulating in South Africa from 2005–2009 are provided in Table 1.

#### Influenza A(H1N1)pdm09

Of the 1,589 patients with ILI enrolled, 496 (31%) tested positive for influenza A(H1N1)pdm09 virus. Vaccination status was known for 1,496 (94%) patients. Of these, 54 (3.6%) received seasonal influenza vaccine. Vaccine coverage was higher in those aged ≥50 (8.6%, 12/140) than in the <50 (3.1%, 42/1,356) age group. The detection rate was higher in those aged <50 (33%, 446/1356) than in the ≥50 (13%, 18/140) years age group. The age-adjusted VE estimate was −6.4% (95% CI: −93.5% to 43.3%) (Table 2).

**Table 2:** Vaccination coverage* and vaccine effectiveness by year, Viral Watch, South Africa: 2005–2009.

Year, season	Vaccination coverage				
	Overall	Cases % (n/N)	Controls % (n/N)	Crude VE (95% CI)	Age-adjusted VE(95% CI)
*Seasonal influenza*					
**2005**	4.1 (49/1199)	2.7 (15/558)	5.3 (34/641)	**50.9 (9.8, 74.2)**	**48.6 (4.9, 73.2)**
2006	5.1 (54/1055)	5.5 (26/476)	4.8 (28/579)	−13.1 (−96.5, 35.9)	−14.2 (−99.7, 34.8)
2007	4.2 (40/957)	3.6 (16/446)	4.7 (24/511)	24.5 (−43.9, 61.2)	12.0 (−70.4, 55.4)
**2008**	3.7 (32/858)	1.5 (4/261)	4.7 (28/597)	**68.3 (15.1, 90.6)**	**67.4 (12.4, 90.3)**
2009	3.4 (59/1737)	2.8 (20/716)	3.8 (39/1021)	27.6 (−24.2, 58.9)	29.6 (−21.5, 60.1)
*A(H1N1)pdm09*					
2009	3.6 (54/1496)	3.4 (16/464)	3.7 (38/1 032)	6.6 (−67.6, 49.7)	−6.4 (−93.5, 43.3)

**Statistically significant values in bold.**

* The proportion of individuals who received at least one dose of influenza vaccine in the relevant period.

Age groups: <50 and ≥50 years.

## Discussion

This article is among the first to describe influenza vaccine effectiveness on the African Continent. We demonstrated influenza vaccine effectiveness against seasonal influenza in two (2005 and 2008) of the five years surveyed where the seasonal A (H1N1) subtype was dominant. However, we did not demonstrate significant VE during the years when the H3N2 subtype was dominant. This is similar to what was reported in other settings when a strain mismatch of the A(H3N2) vaccine component was identified [17], [22]. In 2006 and 2007 there was a mismatch between the H3N2 vaccine composition strains and circulating viruses. The molecular characterization of representative influenza A (H3N2) isolates circulating in South Africa showed an extensive genetic drift from the vaccine component strains A/California/7/04 (H3N2)–like virus, A/Wisconsin/67/05 (H3N2)-like virus respectively [23]–[24]. In 2006 the circulating influenza A (H3N2) viruses were related to the A/Wisconsin/67/05 (H3N2)-like virus while in 2007 were related to the A/Brisbane/10/07 (H3N2)–like virus respectively [23]–[24]. In 2009, the majority of the H3N2 isolates were characterized by antigenic distances of ≥3.25 when compared to the vaccine component strain (Unpublished data – Treurnicht F *et al* In prep). Nonetheless, several studies have shown that influenza vaccines can afford cross-protection against non-matching circulating strains [25].

In addition, as expected, there was no significant protective effect of the seasonal influenza vaccine against the influenza A(H1N1)pdm09 virus strain. This finding has been reported in other countries and settings [26]. It is known that vaccine efficacy depends on the match between the vaccine composition and the circulating strain [3]. In 2009, a new influenza virus strain emerged, making communities vulnerable to influenza as this strain was not included in the Southern hemisphere 2009 seasonal influenza vaccine. When adjusting for age, VE estimates decreased; similar findings have been reported in other settings [17], [22]. While vaccination with seasonal influenza vaccines may induce some increase in antibody response to pandemic virus in adults aged <60 years, increases are not observed in older individuals [27]–[28]. This is likely as a result of preexisting cross-reactive antibodies in this age group. It has also been suggested that seasonal influenza infection may protect against infection with the pandemic strain [29].

Although influenza is a vaccine-preventable disease, the uptake of influenza vaccine in developing countries is poor. Even in industrialized countries, large proportions of high risk groups do not receive influenza vaccine [30]. In South Africa, influenza vaccine coverage among ILI patients seen at Viral Watch sentinel sites, which includes mainly private general practitioners, was very low (average 4%) in all five influenza seasons. While coverage was generally higher amongst the elderly, it remained below 10%. Unfortunately as data on high risk groups were not available, coverage in these important risk populations could not be assessed. Challenges in acquiring data on the epidemiology and burden of influenza continue. However, advances had been made in understanding the epidemiology, burden and seasonality of influenza as illustrated by surveillance activities being done in the continent [2], [15], [31]. Efforts to increase and maintain high vaccination coverage should be emphasized especially to groups at high risk for severe and complicated disease.

There was a significant difference in the age distribution of case-patients between those with pandemic and seasonal influenza. Patients infected with A(H1N1)pdm09 virus were younger than those with seasonal influenza. This is similar to what has been described in other settings [32]–[34]. In our study older children and young adults aged 5–24 years comprised 68% of patients infected with A(H1N1)pdm09 virus (only one was aged >65 years). This might be as a result of some residual protective immunity among older adults from past exposures to the A H1N1 virus as well as potential bias in specimen collection [35]–[36].

In South Africa, influenza epidemics were experienced from May to September and peaked during winter months (June – August) in all five pre-pandemic influenza seasons. This is expected as South Africa experiences a temperate climate. However, in 2009, South Africa experienced two apparent influenza peaks, the first corresponding to the expected annual seasonal influenza season followed by a second and distinct wave that was dominated by influenza A(H1N1)pdm09 and extended to the spring months. This is in contrast to what has been experienced in other temperate southern hemisphere countries, where only one influenza peak predominated by A(H1N1)pdm09 virus was observed [37].

Our study has several limitations. First, we could not assess the severity of cases or influenza-related mortality as the Viral Watch system surveys only outpatients with ILI. Second, data presented here relate only to patients who presented with ILI at sentinel sites from whom specimens were taken, it is not representative of all influenza infections in South Africa. However, it does provide information on the epidemiology of ILI in seasonal and pandemic influenza years which may be of value to the health care system. Third, vaccination status was determined from data collection forms as reported by practitioner; we did not verify these reports ourselves. Fourth, in the VE analysis we could not control for potential confounders such as underlying medical conditions as these data were not available for the years surveyed. We did control for differences in age distribution, which has been shown to be the most important potential confounder of vaccine effectiveness estimates, however due to limitations in numbers of cases we were only able to stratify by two age bands [16], [38]. Fifth, due to low vaccine coverage and low case numbers in some years we may have been under-powered to determine low and subtype-specific VE. Lastly, since the HAI is less sensitive than the PCR we may have some false negatives in some years resulting in some cases being included as controls. This may have some effect on the VE estimate, probably overestimating VE during the years 2005–2007 when mainly HAI was used for the identification of influenza.

In conclusion, this longstanding influenza surveillance programme was able to monitor the circulation of seasonal and pandemic influenza when it occurred in the country and allowed for the estimation of annual VE. Efforts should be made to increase vaccination coverage and improve the data collection tools (e.g. including the collection of information on underlying medical conditions and other possible confounders) to allow for proper adjustment of VE estimates. Feedback to clinicians on vaccine effectiveness will encourage them to participate in the influenza surveillance programme as well as to vaccinate. In addition, vaccine effectiveness data may be useful to countries in the Northern hemisphere if similar strains circulate in their upcoming influenza season.
